# Learning patient-level prediction models across multiple healthcare databases: evaluation of ensembles for increasing model transportability

**DOI:** 10.1186/s12911-022-01879-6

**Published:** 2022-05-25

**Authors:** Jenna Marie Reps, Ross D. Williams, Martijn J. Schuemie, Patrick B. Ryan, Peter R. Rijnbeek

**Affiliations:** 1 Janssen Research and Development, Raritan, NJ USA; 2 Department of Medical Informatics, Erasmus University Medical Center, Rotterdam, The Netherlands

**Keywords:** Ensemble learning, Model transportability, Prognostic model, Observational data, Patient-level prediction

## Abstract

**Background:**

Prognostic models that are accurate could help aid medical decision making. Large observational databases often contain temporal medical data for large and diverse populations of patients. It may be possible to learn prognostic models using the large observational data. Often the performance of a prognostic model undesirably worsens when transported to a different database (or into a clinical setting). In this study we investigate different ensemble approaches that combine prognostic models independently developed using different databases (a simple federated learning approach) to determine whether ensembles that combine models developed across databases can improve model transportability (perform better in new data than single database models)?

**Methods:**

For a given prediction question we independently trained five single database models each using a different observational healthcare database. We then developed and investigated numerous ensemble models (fusion, stacking and mixture of experts) that combined the different database models. Performance of each model was investigated via discrimination and calibration using a leave one dataset out technique, i.e., hold out one database to use for validation and use the remaining four datasets for model development. The internal validation of a model developed using the hold out database was calculated and presented as the ‘internal benchmark’ for comparison.

**Results:**

In this study the fusion ensembles generally outperformed the single database models when transported to a previously unseen database and the performances were more consistent across unseen databases. Stacking ensembles performed poorly in terms of discrimination when the labels in the unseen database were limited. Calibration was consistently poor when both ensembles and single database models were applied to previously unseen databases.

**Conclusion:**

A simple federated learning approach that implements ensemble techniques to combine models independently developed across different databases for the same prediction question may improve the discriminative performance in new data (new database or clinical setting) but will need to be recalibrated using the new data. This could help medical decision making by improving prognostic model performance.

**Supplementary Information:**

The online version contains supplementary material available at 10.1186/s12911-022-01879-6.

## Background

Big observational healthcare databases, such as insurance claims data or electronic healthcare records, often contain data on large and diverse populations. One area where these datasets may benefit healthcare is in the application of machine learning to develop prognostic models. Prognostic models aim to predict a patient’s risk of experiencing some future event (e.g., cardiovascular illnesses) [1] based on their current and historic health. In general, a prognostic task can be decomposed into three parts, the target population/index, the outcome, and the time-at-risk [2]. The target population is the set of patients for whom you attempt to predict the risk of some future outcome and the index is the point in time you want to make the prediction. The outcome is the medical event you want to predict, and the time-at-risk is the time interval (relative to the index) you want to predict the outcome occurring within. Prognostic models are learned from observational healthcare databases by finding patients in the database who historically match the target population, determining features such as age, gender, and medical history at index for each patient and then observing whether they had the outcome during the time-at-risk. Supervised learning, such as binary classification, is then applied to learn the differences between the people who had the outcome during the time-at-risk vs the people who did not. Often the aim is to develop a model using the historical data but apply the model to current patients to calculate a probability of whether they will have the outcome during the future time-at-risk. Such models could improve healthcare by informing medical decision making, but only if these models perform sufficiently well when implemented in their intended setting. For example, a model intended to be used by a family medicine doctor to help them decide which patients should be given preventative medicine may be developed using a large insurance claims database but needs to transport well into the family medicine setting. The performance in a new database (transportability) of a model is initially assessed by externally validating a model across diverse datasets with different patient case mixes [2, 3]. It is common for a model’s performance to deteriorate when transported to a different database [2]. The deterioration in performance may be due to the model or the differences between the development and validation populations [4]. A model that transports well to other databases is much more valuable in clinical practice. The question is how to best develop models with high transportability?

Big observational healthcare datasets only contain a sample of the population. This is frequently a non-random sample, for example the data may over sample (or only contain) certain ethnicities, genders, ages or patients with low/medium/high wealth. If a database used to develop a prognostic model contains a non-random sample of the target population then this will most likely negatively affect its performance if applied on the full population. However, different datasets, with varying patient case mixes, may give diverse perspectives when developing prognostic model for the same prediction task. Learning models across different healthcare datasets (e.g., a US insurance claims database, a UK primary care database and a US electronic healthcare record database) may lead to more transportable models.

There are three potential ways to learn across multiple data sets. The first is the combine the data together into a centralized location (centralized data sharing) and then learn the model using the combined data. The first option is generally limited as sharing patient-level data between researchers is often not possible due to privacy restrictions and therefore it is not possible to train a single model using the combination of different datasets. The second approach is to use federated learning [5–8] where a server communicates between datasets held in different locations to iteratively learn a model by communicating multiple times with each dataset.

The second option has been illustrated to perform similarly to models trained from centralized data sharing [9] but has technical issues that still require improvement [10]. For example, data heterogeneity [10, 11], privacy concerns [9, 11] and the ability to communicate or the number of communication rounds required all limit the applicability of federated learning. Some federated learning methods can reduce the number of communication rounds but still generally require > 30 rounds of communication [12]. Some generalized linear models federated learning methods exist that only require access to each database once, termed ‘one-shot distributed algorithms’[8]. However, the one-shot approach is not currently possible for most machine learning models. The third approach is to combine models developed separately using separate datasets. The third option is most feasible, as it is possible for researchers to easily share prognostic models they develop using their own data and these models could be combined via ensemble techniques (ensemble modeling is the common machine learning approach used to combine binary classification models). This prompts the question; can we implement the third option and combine models developed using diverse datasets to improve model transportability in new data (e.g., in a clinical setting)?

Ensemble learning is the process of producing multiple models, potentially pruning the set of models, and then combining the remaining models [13]. Often the ensemble increases both model accuracy and performance stability compared to any single classifier [14]. Ensembles either combine homogeneous models (same learning algorithm) or heterogeneous models (different learning algorithms). Homogeneous ensembles use the same learning algorithm but modify the perspective by using different training data (e.g., different instances, different features or by adding noise), different metrics or using different model settings (e.g., hyper-parameter values). Heterogeneous models take a different perspective as each learning algorithm makes different assumptions about the data. Combining the models is often done by fusing the models [15], stacking [16] or using a mixture of experts [17]. Examples of simple fusing models include (1) majority vote also known as ‘bagging’ [18], (2) calculating the mean predicted probability value across models or (3) weighted mean of the models’ predicted probabilities based on performance measures. Weighing each model’s predicted probability based on the model’s performance is better than taking the mean of all the models’ predicted probabilities when the models’ performances differ (e.g., one model is better than the others) [15]. A mixture of experts is similar to weighted mean fusion but instead of using universal weights across the instances, the weights are assigned per instance [18]. These ensembles are considered independent ensemble frameworks, as the models are trained independently and then combined [14]. A more advanced independent ensemble framework is known as ‘stacking’. Stacking is a meta-combination method that uses the set of models’ predicted probabilities as features and trains a new model that learns to predict the outcome using these prediction features [16]. A limitation of stacking is that it requires additional labelled data to learn how to best combine the individual models. Alternatively, ‘dependent ensemble’ frameworks train models sequentially and each model depends on the output of the prior model [14]. Boosting is a dependent fusion ensemble framework as models are sequentially trained, and weights are assigned to the objective function of each model during training based on prior models’ mistakes [19]. The above examples are just a selection of the commonly used combination methods and there are numerous other ways to combine the models [14].

### Objective

This paper aims to determine whether prognostic model ensembles that combine regularized logistic regression models independently developed across different healthcare databases perform better in new data (more transportable) than each individual database prognostic model (single dataset model). A model with improved transportability is likely to also perform better when used clinically for decision making.

## Methods

The Observational Health Data Science and Informatics (OHDSI) PatientLevelPrediction framework is used throughout this paper [2] for developing prognostic models using observational healthcare data.

### Databases

Four US claims and an EHR databases are explored, see Table 1.

**Table 1:** Summary of the five databases used in this study

Name	Type	Description	Start	End	Size (million lives)
IBM Medicare Supplemental Beneficiaries (MDCR)	US Claims	Patients aged 65 or older with supplemental healthcare	2000–01–01	2019–12–31	10
IBM Medicaid (MDCD)	US Claims	Patients with government subsidized healthcare	2006–01–01	2018–12–31	28
Optum® De-Identified Clinformatics® Data Mart Database (Optum Claims)	US Claims	Patients of all ages	2000–05–01	2019–12–31	84
IBM Commercial Claims and Encounters (CCAE)	US Claims	The patients in this database are aged 65 or younger. They are employees who receive health insurance through their employer and their dependents	2000–01–01	2019–12–31	152
Optum® de-identified Electronic Health Record Dataset (Optum EHR)	US EHR	Patients of all ages	2006–01–01	2019–03–31	96

The five databases in this study contain retrospectively collected deidentified data. The use of IBM and Optum databases were reviewed by the New England Institutional Review Board (IRB) and were determined to be exempt from broad IRB approval.

All datasets used in this paper were mapped into the OHDSI Observational Medical Outcomes Partnership Common Data Model (OMOP-CDM) version 5 [20]. The OMOP-CDM was developed to enable researchers with diverse datasets to have a standard database structure. This enables analysis code and software to be shared among researchers which facilitates external validation of prediction models.

### Prediction problem

As an example, the problem: “Amongst patients with pharmaceutically-treated depression, which patients will develop < an outcome > during the 1-year time interval following the start of the depression episode?” is investigated.

The target population of pharmaceutically treated depressed patients is defined as: patients with a condition record of major depressive disorder and the index date was the first record date. Inclusion criteria are:

- Antidepressant recorded within 30 days before to 30 days after the target population index date
- No history of psychosis
- No history of dementia
- No history of mania
-  >  = 365 days prior observation

Twenty-one models predicting 21 different outcomes occurring for the first time between 1 day after index until 1 year after index are developed. The 21 outcomes are: acute liver injury, acute myocardial infarction, alopecia, constipation, decreased libido, delirium, diarrhea, fracture, gastrointestinal hemorrhage, hyponatremia, hypotension, hypothyroidism, insomnia, nausea, seizure, stroke, sudden cardiac death, suicide and suicidal ideation, tinnitus, ventricular arrhythmia and vertigo.

The above definition of prediction problem is the same as used in reference [2].

In this study a random sample of 500,000 patients from the target population (> 1 million patients in Optum claims, > 2 million patients in Optum EHR and > 2 million in CCAE) were used throughout the study. Two database (MDCR and MDCD) has less than 500,000 patients, so no sampling was done. This improved the efficiency of model development and also resulted in some low outcome counts, enabling the investigation into whether the outcome count impacts the ensemble performance.

### Labelled data

We constructed labelled datasets for each database and outcome pair. For the nth patient in database k we used one-hot-encoding to create binary features indicating the presence of any medical condition or drug recorded prior to index (first record of major depressive disorder) and extracted the patient’s gender and age at index (in 5-year bins). Full feature construction details can be found in Additional file 1: Appendix A. Let ${\mathbf{x}}_{\mathbf{n}}^{\mathbf{k}}$ represent the feature vector for thenth patient in database k. Labels were determined per outcome, with ${\mathrm{y}}_{\mathrm{nj}}^{\mathrm{k}}$ corresponding to the presence (${\mathrm{y}}_{\mathrm{nj}}^{\mathrm{k}}=1)$ or absence (${\mathrm{y}}_{\mathrm{nj}}^{\mathrm{k}}=0)$ of outcome j in the year after index for patient n in database k. This resulted in 105 labelled datasets ${\{({\mathbf{x}}_{\mathbf{n}}^{\mathbf{k}},{\mathrm{y}}_{\mathrm{nj}}^{\mathrm{k}})\}}_{\mathrm{n}}$.

### Statistical analysis

#### Binary classifiers (Level 1 models)

For each database and outcome, a regularized logistic regression model with least absolute shrinkage and selection operator (LASSO) penalization was trained [21] using 80% of the data to develop the model and 20% of the data were held out to internally estimate the model performance (test set performance). Three-fold cross validation was applied in the 80% development data to learn the optimal regularization value. The LASSO logistic regression implementation we used automatically searches for the variance (the regularization parameter), starting from a variance of 0.01, that maximizes the model discrimination [21]. The final LASSO logistic regression coefficients were learned with the optimal hyper-parameter set using all of the 80% development data.

Let ${\mathrm{f}}_{\mathrm{ij}}\left(\mathbf{x}\right):{\mathrm{R}}_{\mathrm{m}}\to [\mathrm{0,1}]$ correspond to the Level 1 logistic regression model that was developed using the ith database (database i) to predict the jth outcome (outcome j), where **x** is the m-dimension feature vector for a patient. Given a patient’s feature vector, the Level 1 model developed in database i predicts a value between 0 and 1 that corresponds to the probability that the patient will experience outcome j.

#### Performance evaluation

Internal validation is when a model is developed and evaluated in the same database and external validation is when a model is developed and evaluated in different databases. For both internal and external validation, model discrimination and calibration were calculated. Model discrimination assesses how well a model ranks patients based on risk, this was calculated using the area under the receiver operating curve (AUROC). The AUROC is a ranking measure that corresponds to the probability that if a non-outcome patient was sampled and an outcome patient was sampled, the predicted risk assigned to the outcome patient is greater than the predicted risk assigned to the non-outcome patient. An AUROC of 0.5 corresponds to randomly predicting risk (no discriminative ability) and an AUROC of 1 corresponds to perfect prediction (a higher risk is predicted for all patients who will experience the outcome compared to those who will not). Calibration assesses how closely the predicted risk matches the true risk. For example, if a model is well calibrated, then if 10 patients are assigned a 10% risk, only 1 of them should experience the outcome. In this study, calibration was calculated using calibration-in-the-large [22] which compares the model’s mean predicted risk in the population with the observed risk (a model is considered well calibrated if the mean predicted risk matches the observed risk in the population).

The internal validation of each Level 1 model (test set performance) provides a benchmark performance for the database and outcome pair. The internal validation of each Level 1 model, trained in database k to predict outcome j, was determined by calculating the AUROC and calibration-in-the-large using the predicted risk ${\mathrm{f}}_{\mathrm{kj}}\left({\mathbf{x}}_{\mathbf{n}}^{\mathbf{k}}\right)$ and the true label ${\mathrm{y}}_{\mathrm{nj}}^{\mathrm{k}}$ for each patient in the 20% held out set (test set).

#### Binary ensemble classifiers (Level 2 models)

The ensembles in this study combine the Level 1 models developed in the different databases that predict the same outcome. Generally, an ensemble that predicts outcome j is a function of the N Level 1 models that predict outcome j:

$${\mathrm{f}}_{\mathrm{j}}\left(\mathbf{x}\right)=\mathrm{g}({\{{\mathrm{f}}_{\mathrm{ij}}(\mathbf{x})\}}_{\mathrm{i}\in \{\mathrm{1,2},\dots ,\mathrm{N}\}})$$

Seven different ensemble approaches were investigated to combine the Level 1 models, that predict the same outcome (j) but are trained on N different databases (${\{{\mathrm{f}}_{\mathrm{ij}}\}}_{\mathrm{i}\in \{\mathrm{1,2},\dots ,\mathrm{N}\}}$), using different heuristics.

A weighted fusion ensemble to predict the outcome j combines the Level 1 models by assigning each Level 1 model a weight:

$${\mathrm{f}}_{\mathrm{j}}\left(\mathbf{x}\right)= \sum_{\mathrm{i}}{{\mathrm{w}}_{\mathrm{ij}}\mathrm{f}}_{\mathrm{ij}}(\mathbf{x})$$

where ${\mathrm{w}}_{\mathrm{ij}}$ is the weight assigned to the Level 1 model trained using database i to predict outcome j. We investigated five different fusion ensembles. The simplest fusion is the uniform weighted one that simply takes the mean of the models’ predicted probabilities for each patient. This was chosen due to simplicity, for prognostic models, the simplest model that performs well is often preferred as it is easier to implement. However, we also investigated two performance weighted fusions as prognostic model performance often varies depending on the development dataset and it seems reasonable to give a model with higher internal performance more weight. As AUROC is the most common discrimination metric, we chose this. In general, you expect a model to perform better when applying the model to a new dataset that has a similar case-mix to the development data compared to a different case-mix. This prompted the investigation of weighing each model’s predicted probabilities based on how similar the model’s development population are to the validation population (i.e., when applying an ensemble, weight models developed on similar data more than models developed on different data compared to the application data). Finally, weights based on the similarity between the development population mean age and validation population mean age was investigated because age is often a key predictor in prognostic models, as seen in published dementia models [23]. Datasets often have skewed age distributions (contain younger or older populations compared to the general population). Although age is a candidate predictor in each Level 1 model, if a certain age group is not observed in the database (e.g., CCAE contains no patients aged 65 or higher), it will not be possible for the model to learn the association between the unobserved ages and the outcome. Consequently, an ensemble that assigns a higher weight to models developed using populations that are similar in age to the application population may perform better.

In this study different weighting heuristics are investigated:

1. Mean Ensemble (**mean**)—for a patient, their predicted risk is the mean of the predicted risks of the included Level 1 classifiers (equal weighting so ${\mathrm{w}}_{\mathrm{ij}}$ = 1/N, where N is the number of models being combined)
2. AUROC Ensemble normalized weights (**auc1**)—for a patient, their predicted risk is a weighted mean of the predicted risks of the included Level 1 models, where each Level 1 model’s weight is based on the model’s internal area under the receiver operating characteristic curve (AUROC) that was calculated in the 20% held out data. The weights are scaled relative to an AUROC of 0.5 and normalized to ensure the total weight across models was 1 (AUROC performance weighting so ${\mathrm{w}}_{\mathrm{ij}}= \frac{{|\mathrm{AUROC}}_{\mathrm{ij}}-0.5|}{\sum_{\mathrm{k}}{|\mathrm{AUROC}}_{\mathrm{kj}}-0.5|}$), where AUROC_ij_ is the internal AUROC value for the Level 1 model developed in database i to predict outcome j.
3. AUROC Ensemble unnormalized weights (**auc2**)—similar to 2) a patient’s risk is a weighted mean of the predicted risks of the included Level 1 models, where each Level 1 model’s weight is based on the model’s internal AUROC. The weights are scaled between 1 for perfect discrimination and -1 for models that predict the opposite labels perfectly (${\mathrm{w}}_{\mathrm{ij}}= \frac{{\mathrm{AUROC}}_{\mathrm{ij}}-0.5}{0.5}$), where AUROC_ij_ is the internal AUROC value for the Level 1 model developed in database i to predict outcome j.
4. Similarity Weighted Ensemble (**sim**)- for a patient, their predicted risk is a weighted mean of the predicted risks of the included Level 1 models, but weights are based on how similar the Level 1 model’s development population mean value for each predictor are compared to the population that the patient is in. The cosine similarity metric was used for the two vectors containing the mean values in the patient’s dataset and the Level 1 model’s development data (case mix similarity weighting ${\mathrm{w}}_{\mathrm{ij}}=\frac{{\mathrm{cosine}(\mathbf{d}, \mathbf{d}}_{\mathbf{i}})}{\sum_{\mathrm{k}}{\mathrm{cosine}(\mathbf{d}, \mathbf{d}}_{\mathbf{k}})}$) where **d** is an m-dimensional vector corresponding to the mean values of the features included in model ${\mathrm{f}}_{\mathrm{ij}}$ in the database the ensemble is being applied to and **d**_**i**_ is an m-dimensional vector corresponding to the mean values of the features included in model ${\mathrm{f}}_{\mathrm{ij}}$ in database i.
5. Age Weighted Ensemble (**age**)– for a patient, their predicted risk is a weighted mean based on how similar the model development data population mean age was compared to the patient’s population mean age (case age similarity weighting ${\mathrm{w}}_{\mathrm{ij}}= \frac{{\mathrm{d}(\mathrm{\mu}_\mathrm{age},\mathrm{ age}}_{\mathrm{i}})}{\sum_{\mathrm{k}}{\mathrm{d}(\mathrm{\mu}_\mathrm{age},\mathrm{ age}}_{\mathrm{k}})}$), where *μ*_age_ is the mean age in years of the patients in the dataset the model is being applied to, age_i_ is the mean age of the patients in database i and  ${\mathrm{d}(\mathrm{\mu}_\mathrm{age},\mathrm{ age}}_{\mathrm{i}}) = 1/(1 + \left|\mathrm{\mu}_{\mathrm{age}}-{\mathrm{age}}_{\mathrm{i}}\right|)$.
  The mixture of expert ensembles ${\mathrm{f}}_{\mathrm{j}}\left(\mathbf{x}\right)$ use the equation:${\mathrm{f}}_{\mathrm{j}}\left(\mathbf{x}\right)= \sum_{\mathrm{i}}{{\mathrm{g}}_{\mathrm{ij}}(\mathbf{x})\mathrm{f}}_{\mathrm{ij}}(\mathbf{x})$ where ${\mathrm{g}}_{\mathrm{ij}}$ is the gating function value for Level 1 model developed in database i to predicted outcome j. We used age as to determine which model is most suitable for a patient as differences in age between the development and validation datasets often impact performance.
6. Age Mixture of Experts Ensemble (**ageME**) – for a patient, their predicted risk is calculated using the Level 1 model developed using a population with a mean age that most closely matches the patient’s age, the gating function is:
  $${\mathrm{g}}_{\mathrm{ij}}\left(\mathbf{x}\right)=\left\{\begin{array}{c}1, i \equiv \underset{\mathbf{k}}{\mathbf{min}}{(\mathbf{a}\mathbf{g}\mathbf{e}}_{\mathbf{k}}-\mathbf{a}\mathbf{g}\mathbf{e}) \\ 0, otherwise\end{array}\right.$$
  where ${\mathbf{a}\mathbf{g}\mathbf{e}}_{\mathbf{k}}$ is the mean age in years of the patients in database k and $\mathbf{a}\mathbf{g}\mathbf{e}$ is the age in years of the patient whose risk is being calculated.
  Stacking ensembles involved training a Level 2 model that uses the Level 1 model predicted probabilities as features. Stacking ensembles were investigated as they have the advantage that they may be able to use small amounts of labelled data from the application dataset to learn how to weight each model. It would also be possible to learn the stacking model using one of the development datasets, however this would reduce the number of Level 1 models in the ensemble and was not explored in this study.
7. Stacking ensemble –a logistic regression model was trained as the Level 2 model that used the predicted risk from each Level 1 model as predictors (effectively this learned the Level 1 model weightings). The stacking ensemble requires labelled data in the validation dataset whereas the other ensembles do not require this. As it is often not possible to get large amounts of labelled data in the validation dataset or application dataset, it was investigated how well the stacking ensemble would do if i) only 1,000 patients (**s|1000**), ii) only 10,000 patients (**s|10,000**) and iii) all available patients (**s|All**) were used to learn the weightings.

#### Model transportability

**Fig. 1:**
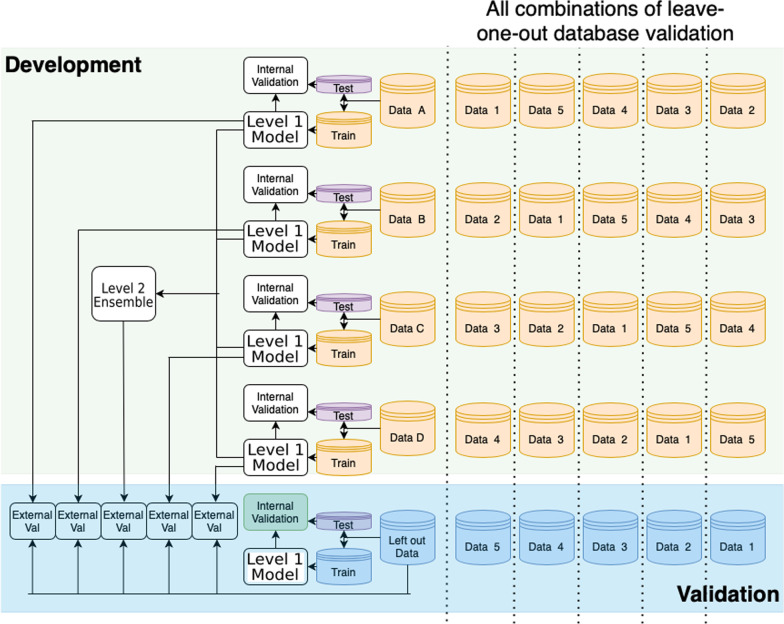
The leave-one-database-out design used to evaluate the transportability of the Level 1 models trained using a single database and the Level 2 ensembles that combine multiple Level 1 models. The figure shows that five different combinations were used, where four of the five databases were used to develop the models and the final database was used to fairly evaluate the transportability of the models. In addition, a model was trained using the left-out database to calculate the internal validation that could be considered the ‘internal benchmark’ performance for the database given sufficient training data. We compared how similar the external validation of each model was with the ‘internal benchmark’

For each ensemble model a leave-one-database out approach was used to estimate external validation when the ensemble was transported to new data. Figure 1 illustrates the leave-one-database out approach. For example, to estimate the mean fusion ensemble performance in predicting insomnia when externally validated on MDCR, the Level 1 models trained on the MDCD, CCAE, Optum Claims and Optum EHR to predict insomnia were applied to each patient in MDCR and then the mean of the patient’s predicted risks across the four Level 1 models was calculated per patient. The mean fusion ensemble predictions are then validated using the ground truth labels in the left-out database where it was known which patients experienced insomnia. This was repeated five times by leaving each database out once.

Denoting the set of feature and label pairs in database k for outcome j as: ${\{({\mathbf{x}}_{\mathbf{n}}^{\mathbf{k}},{\mathrm{y}}_{\mathrm{nj}}^{\mathrm{k}})\}}_{\mathrm{n}}$, the vector of predicted risks in database k for outcome j using the mean fusion ensemble across N database models excluding the database k model is:

$${\mathbf{p}\mathbf{r}\mathbf{e}\mathbf{d}}_{\mathbf{j}}^{\mathbf{k}}= (\sum_{\mathrm{i}\ne \mathrm{k}}\frac{{\mathrm{f}}_{\mathrm{ij}}\left({\mathbf{x}}_{1}^{\mathbf{k}}\right)}{\mathrm{N}},\sum_{\mathrm{i}\ne \mathrm{k}}\frac{{\mathrm{f}}_{\mathrm{ij}}\left({\mathbf{x}}_{2}^{\mathbf{k}}\right)}{\mathrm{N}}, \dots , \sum_{\mathrm{i}\ne \mathrm{k}}\frac{{\mathrm{f}}_{\mathrm{ij}}\left({\mathbf{x}}_{\mathbf{m}}^{\mathbf{k}}\right)}{\mathrm{N}} )$$

The ground truth in database k is:

$${\mathbf{t}\mathbf{r}\mathbf{u}\mathbf{t}\mathbf{h}}_{\mathbf{j}}^{\mathbf{k}}=({\mathrm{y}}_{1\mathrm{j}}^{\mathrm{k}}, {\mathrm{y}}_{2\mathrm{j}}^{\mathrm{k}}, \dots , {\mathrm{y}}_{\mathrm{mj}}^{\mathrm{k}})$$

The external AUROC and calibration metrics for the mean fusion ensemble applied to database k for outcome j is then calculated by comparing the predictions and ground truth labels.

$${\mathrm{externalAUROC}}_{\mathrm{j}}^{\mathrm{k}}=\mathrm{AUROC}({\mathbf{p}\mathbf{r}\mathbf{e}\mathbf{d}}_{\mathbf{j}}^{\mathbf{k}},{\mathbf{t}\mathbf{r}\mathbf{u}\mathbf{t}\mathbf{h}}_{\mathbf{j}}^{\mathbf{k}})$$

In general, the predictions for all Level 1 models and Level 2 ensemble models when transported to database k are:

$${\mathbf{l}\mathbf{e}\mathbf{v}\mathbf{e}\mathbf{l}1\mathbf{P}\mathbf{r}\mathbf{e}\mathbf{d}}_{\mathbf{j}}^{\mathbf{k}}= \left({\mathrm{f}}_{\mathrm{ij}}\left({\mathbf{x}}_{1}^{\mathbf{k}}\right), {\mathrm{f}}_{\mathrm{ij}}\left({\mathbf{x}}_{2}^{\mathbf{k}}\right), \dots ,{\mathrm{f}}_{\mathrm{ij}}\left({\mathbf{x}}_{\mathbf{m}}^{\mathbf{k}}\right)\right),\mathrm{ where \quad i}\ne \mathrm{k}$$

$${\mathbf{e}\mathbf{n}\mathbf{s}\mathbf{e}\mathbf{m}\mathbf{b}\mathbf{l}\mathbf{e}\mathbf{P}\mathbf{r}\mathbf{e}\mathbf{d}}_{\mathbf{j}}^{\mathbf{k}}=(\mathrm{g}\left({\left\{{\mathrm{f}}_{\mathrm{ij}}\left({\mathbf{x}}_{1}^{\mathbf{k}}\right)\right\}}_{\mathrm{i}\ne \mathrm{k}}\right),\mathrm{ g}\left({\left\{{\mathrm{f}}_{\mathrm{ij}}\left({\mathbf{x}}_{2}^{\mathbf{k}}\right)\right\}}_{\mathrm{i}\ne \mathrm{k}}\right), \dots ,\mathrm{ g}\left({\left\{{\mathrm{f}}_{\mathrm{ij}}\left({\mathbf{x}}_{\mathbf{m}}^{\mathbf{k}}\right)\right\}}_{\mathrm{i}\ne \mathrm{k}}\right))$$

To put the performance of the Level 1 and Level 2 models (that do not used database k) into context, the ‘internal benchmark’ performance in database k was estimated. The ‘internal benchmark’ is defined as the internal validation performance (using a 20% test set ${\{({\widehat{\mathbf{x}}}_{\mathbf{i}}^{\mathbf{k}},{\widehat{\mathrm{y}}}_{\mathrm{ij}}^{\mathrm{k}})\}}_{\mathrm{i}}$) of the Level 1 model developed in database k:

$${\mathbf{i}\mathbf{n}\mathbf{t}\mathbf{e}\mathbf{r}\mathbf{n}\mathbf{a}\mathbf{l}\mathbf{P}\mathbf{r}\mathbf{e}\mathbf{d}}_{\mathbf{j}}^{\mathbf{k}}= ({\mathrm{f}}_{\mathrm{kj}}\left({\widehat{\mathbf{x}}}_{1}^{\mathbf{k}}\right),{\mathrm{f}}_{\mathrm{kj}}\left({\widehat{\mathbf{x}}}_{2}^{\mathbf{k}}\right),{\dots ,\mathrm{f}}_{\mathrm{kj}}\left({\widehat{\mathbf{x}}}_{\mathbf{t}}^{\mathbf{k}}\right))$$

$${\mathbf{t}\mathbf{r}\mathbf{u}\mathbf{t}\mathbf{h}\mathbf{T}\mathbf{e}\mathbf{s}\mathbf{t}}_{\mathbf{j}}^{\mathbf{k}}=({\widehat{\mathrm{y}}}_{1\mathrm{j}}^{\mathrm{k}}, {\widehat{\mathrm{y}}}_{2\mathrm{j}}^{\mathrm{k}}, \dots , {\widehat{\mathrm{y}}}_{\mathrm{tj}}^{\mathrm{k}})$$

The internal AUROC in database k for outcome j is then:

$${\mathrm{internalAUROC}}_{\mathrm{j}}^{\mathrm{k}}=\mathrm{AUROC}({\mathbf{i}\mathbf{n}\mathbf{t}\mathbf{e}\mathbf{r}\mathbf{n}\mathbf{a}\mathbf{l}\mathbf{P}\mathbf{r}\mathbf{e}\mathbf{d}}_{\mathbf{j}}^{\mathbf{k}}, {\mathbf{t}\mathbf{r}\mathbf{u}\mathbf{t}\mathbf{h}\mathbf{T}\mathbf{e}\mathbf{s}\mathbf{t}}_{\mathbf{j}}^{\mathbf{k}})$$

Given sufficient data, the internal performance of a model can be considered the upper bound of achievable performance (conditional on the same features being available to internal and external model development). If a model transported to new data has an external performance close to the internal performance of a model developed using the data, then this can be considered to have transported well. Consequently, to determine how well a model transports the difference in performance between the internal validation AUROC of the Level 1 model trained using the left-out database, database k, and the external validation AUROC of models when applied to the left-out database k was calculated:

$${\mathrm{AUROC}\_\mathrm{difference}}_{\mathrm{j}}^{\mathrm{k}}= {\mathrm{externalAUROC}}_{\mathrm{j}}^{\mathrm{k}}-{\mathrm{internalAUROC}}_{\mathrm{j}}^{\mathrm{k}}$$

where ${\mathrm{externalAUROC}}_{\mathrm{j}}^{\mathrm{k}}$ is the performance of the model in database k (trained without dataset k) in predicting outcome j and ${\mathrm{internalAUROC}}_{\mathrm{j}}^{\mathrm{k}}$ is the Level 1 model predicting outcome j trained in database k’s performance on the 20% test set. To show how well each model transports in general, box plots were created to show the distribution of ${\mathrm{AUROC}\_\mathrm{difference}}_{\mathrm{j}}^{\mathrm{k}}$ across the different outcomes and databases. Distributions centered around 0 indicate excellent transportability and distributions with a small range indicate consistency.

## Results

The data sizes are presented in Table 2 and the database characteristics are displayed in Table 3. The smallest target population was the one extracted from the MDCR database, and this population were older and had higher rates of cancer and cardiovascular issues prior to index. The MDCD target population was the youngest and had the highest rate of obesity recorded in the prior year. In general, the characteristics varied greatly across the datasets, indicating different patient case-mixes. The outcome count was generally greater than 100 except for delirium in MDCD and Optum Claims and Seizure in MDCR and Optum EHR.

**Table 2:** The outcome counts and percentage of target population who develop the outcome during the tine-at-risk

Outcome	CCAE (N ~ 499,678) (%)	MDCR (N ~ 160,956) (%)	MDCD (N ~ 469,302) (%)	Optum EHR (N ~ 499,881) (%)	Optum Claims (N ~ 499,753) (%)
Acute liver injury	14,875 (3.35)	7226 (5.4)	21,654 (5.47)	18,535 (4.18)	18,619 (4.31)
Acute myocardial infarction	1494 (0.3)	935 (0.59)	3800 (0.83)	816 (0.16)	1298 (0.26)
Alopecia	10,672 (2.32)	7569 (5.64)	20,597 (5.2)	16,597 (3.69)	16,571 (3.75)
Constipation	4170 (0.85)	6399 (4.39)	9210 (2.05)	10,192 (2.13)	10,282 (2.16)
Decreased libido	491 (0.1)	1080 (0.69)	905 (0.19)	287 (0.06)	708 (0.14)
Delirium	174 (0.03)	510 (0.32)	86 (0.02)	267 (0.05)	91 (0.02)
Diarrhea	1661 (0.34)	130 (0.08)	785 (0.17)	1210 (0.24)	1603 (0.32)
Fracture	509 (0.1)	963 (0.61)	894 (0.19)	381 (0.08)	758 (0.15)
Gastrointestinal hemorrhage	985 (0.2)	1298 (0.81)	1666 (0.36)	356 (0.07)	1021 (0.2)
Hyponatremia	19,754 (4.65)	7824 (5.95)	33,518 (9.82)	24,043 (5.65)	23,304 (5.67)
Hypotension	380 (0.08)	1153 (0.74)	636 (0.14)	230 (0.05)	683 (0.14)
Hypothyroidism	297 (0.06)	642 (0.4)	1056 (0.23)	162 (0.03)	333 (0.07)
Insomnia	3046 (0.62)	2086 (1.38)	2468 (0.53)	3049 (0.62)	4114 (0.85)
Ischemic stroke all inpatient	3120 (0.64)	1824 (1.19)	2655 (0.57)	2775 (0.56)	4139 (0.85)
Nausea	2722 (0.56)	4071 (2.77)	4033 (0.89)	4368 (0.9)	5846 (1.22)
Open angle glaucoma	6117 (1.33)	3853 (2.83)	5374 (1.22)	8786 (2.03)	9943 (2.33)
Seizure	184 (0.04)	67 (0.04)	307 (0.07)	94 (0.02)	199 (0.04)
Suicide and suicidal ideation	10,221 (2.13)	993 (0.62)	21,518 (5.09)	9957 (2.1)	8063 (1.67)
Tinnitus	2628 (0.53)	4276 (2.87)	5082 (1.12)	6920 (1.44)	7643 (1.62)
Ventricular arrhythmia and sudden cardiac death	20,806 (4.91)	6846 (5.12)	27,233 (6.92)	23,655 (5.6)	23,772 (5.89)
Vertigo	2577 (0.53)	748 (0.47)	2269 (0.49)	2341 (0.48)	2782 (0.57)

CCAE/Optum EHR/Optum claims contained more than 500,000 pharmaceutically treated depressed patients so we sampled 500,000 patients from each of these databases

A small number of the 500,000 patients sampled were excluded because the index date was the last time the patient was observed in the data (so they had no follow-up)

**Table 3:** Characteristics of the target population (patients with depression initiating treatment) per database

	CCAE	MDCD	MDCR	Optum Claims	Optum EHR
Mean age	41	35	75	50	49
Male (%)	30.8	25.9	32.2	31.7	29.2
Mean number outpatient visits in prior year	16.3	31.2	26.8	16.6	32.4
Frequency of patients experiencing condition in prior year					
Pain	0.60	0.74	0.74	0.66	0.57
Anxiety	0.41	0.50	0.28	0.42	0.43
Acute inflammatory disease	0.32	0.36	0.24	0.31	0.18
Neoplastic disease	0.22	0.14	0.46	0.27	0.17
Essential hypertension	0.25	0.31	0.69	0.40	0.37
Obesity	0.11	0.19	0.11	0.13	0.17
Heart disease	0.09	0.14	0.46	0.20	0.18
Diabetes mellitus	0.09	0.14	0.27	0.16	0.16
Urinary tract infectious disease	0.09	0.14	0.16	0.12	0.07
Anemia	0.07	0.12	0.20	0.12	0.11

**Fig. 2:**
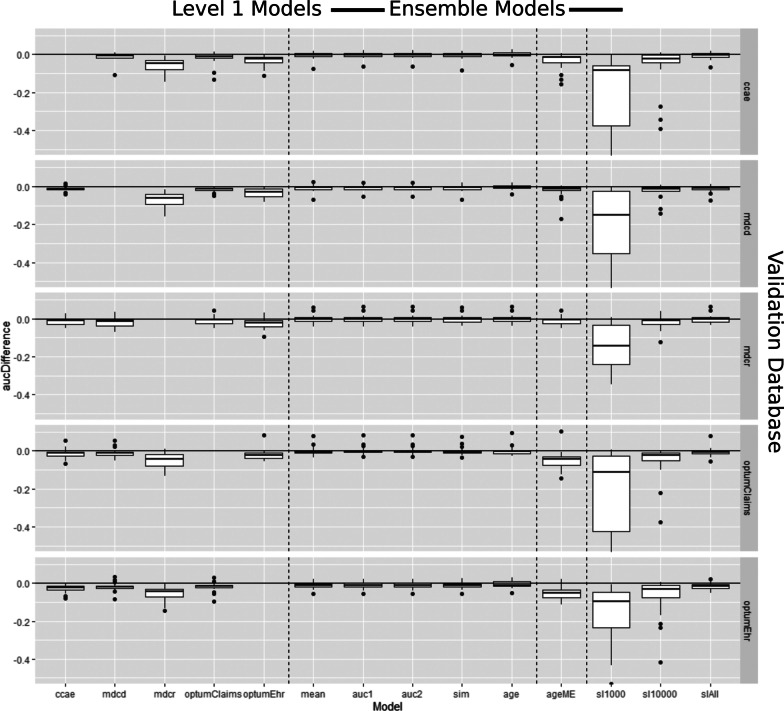
Box plots showing the difference between the external validation AUROC minus the internal validation AUROC per non-ensemble (Level 1 model) and ensemble method (Level 2 model) across the five databases. The rows represent the external database (the database that was excluded from the model/ensemble development) that was used to fairly evaluate the models/ensembles. The x-axis represents the model/ensemble technique. Box plots centered around 0 with a small range indicate highly transportable and consistent external discriminative performance. The dashed vertical lines separate the non-ensembles, the fusion ensembles, the mixture of expert ensembles and the stacking ensembles

Figure 2 presents box plots of the AUROC_differences per Level 1 model (non-ensemble) and Level 2 model (ensemble) when transported to each held out database across the 21 outcomes. A zoomed in version of Fig. 2 can be found in Additional file 2: Appendix B. The non-ensemble box plots show a lower median value and greater range of values compared to the fusion ensembles. The fusion ensembles achieved discriminative performances similar to the ‘internal benchmark’ when transported to new databases (AUROC_difference values close to 0). The age-based mixture of expert and stacking ensembles that used 1,000 or 10,000 labels generally performed worse than the non-ensembles in terms of discrimination when transported. The stacking ensemble using all the labelled data available achieved external AUROC similar to the ‘internal benchmark’ but was not better than the fusion ensembles. The full external validation discrimination performance across the 21 outcomes and 5 databases for the non-ensembles and ensembles are presented in Additional file 3: Appendix C.

**Fig. 3:**
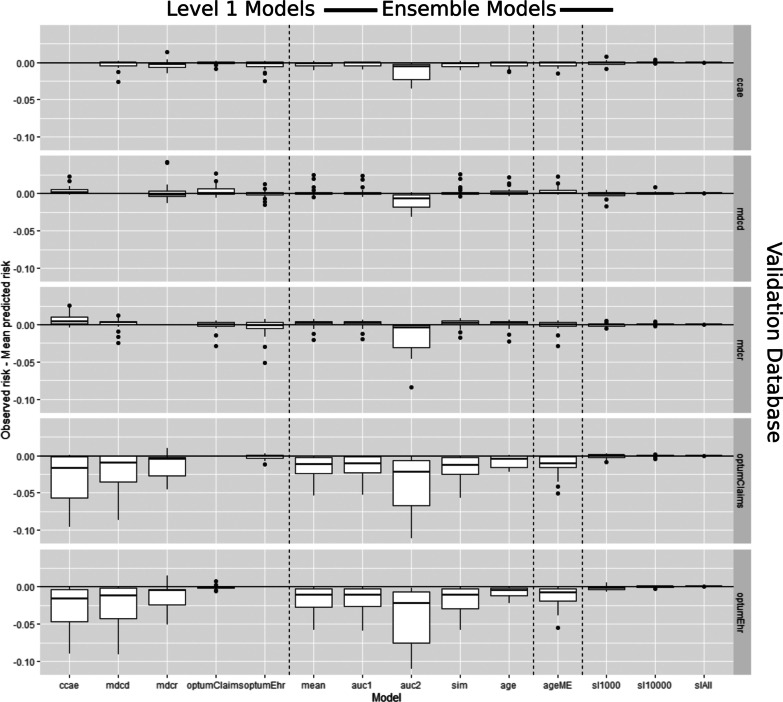
Box plots of calibration-in-the-large (observed risk—mean predicted risk) values for each non-ensemble (Level 1 model) and ensemble (Level 2 model) when externally validated. The rows represent the external database (the database that was excluded from the model/ensemble development) that was used to fairly evaluate the models/ensembles. The x-axis represents the model/ensemble technique. Box plots centered around 0 with a small range indicate excellent external calibration performance. The dashed vertical lines separate the non-ensembles, the fusion ensembles, the mixture of expert ensembles and the stacking ensembles

**Fig. 4:**
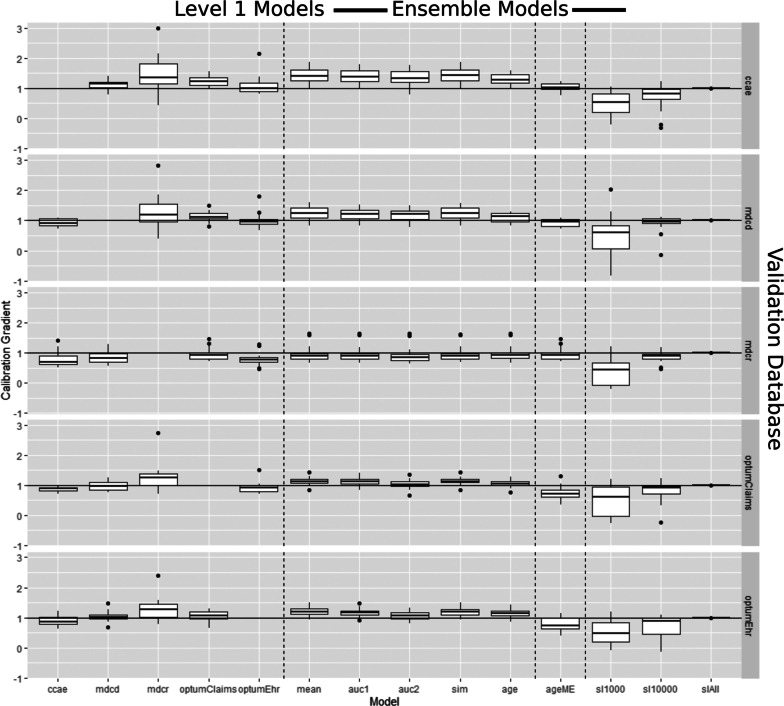
The distribution of calibration gradient (slope) values for each non-ensemble (Level 1 model) and ensemble (Level 2 model) when externally validated. The rows represent the external database (the database that was excluded from the model/ensemble development) that was used to fairly evaluate the models/ensembles. The x-axis represents the model/ensemble technique. Box plots centered around 1 with a small range indicate excellent external calibration performance. The dashed vertical lines separate the non-ensembles, the fusion ensembles, the mixture of expert ensembles and the stacking ensembles

The distribution of calibration in the large values (observed risk—mean predicted risk) is presented in Fig. 3 and the distribution of model calibration gradients (slopes) are presented in Fig. 4. The calibration in the large plots show the difference between the observed risk and the mean predicted risk per Level 2 model (ensemble) or Level 1 model (non-ensemble). A model is well calibrated if the mean predicted risk matches the observed population risk, corresponding to a calibration in the large of 0. Figure 3 show that the mean predicted risks did not often match the observed population risk, except for the stacking ensemble. The calibration gradient (slope) can often indicate overfitting where a model is predicting extremely small risk (close to 0) or large risks (close to 1). If the gradient is much greater than 1, then this indicates overfitting.

## Discussion

The results show that weighted fusion ensembles that combine multiple prognostic models developed in different databases appear to have more stable discriminative performances when transported to new databases compared to the Level 1 (single database) models. However, calibration appears to be an issue for all models that are transported to new databases (except stacking ensembles with sufficient labels).

This study showed that certain ensembles combining models developed independently across difference databases transport better than the Level 1 single database models. The weighted fusion ensembles and stacking ensemble (that used all data) consistently achieved discrimination close to the ‘internal benchmark’ in the new data whereas the Level 1 single models generally performed slightly worse than the ‘internal benchmark’. The Level 1 single database models were also less consistent across outcomes and certain database models did better than others (e.g., Optum claims models transported better than MDCR models). This variability may be due to each database containing diverse patient case-mixes, as seen in Table 3. The ensembles can combine the perspectives of the Level 1 models trained with different populations making them more robust to new populations. The calibrations of the transported models were generally poor, except the stacking model (using all data) as this used labelled data so was effectively recalibrated. If all the Level 1 single database models are mis-calibrated, then it makes sense that any ensemble combining them would also be mis-calibrated. This highlights the importance of model recalibrating before implementing them in new patient populations. It may be possible to recalibrate without labelled data by changing the intercept based on how common the outcome is in the target population the model is being applied to. If labels are available for some patients, then standard recalibration techniques can be implemented. The auc2 fusion ensemble visually had the worse calibration. This is likely due to the weights being between -1 and 1 and not being normalized (total weights did not sum to 1). This highlights that not normalizing the fusion weights can have a large impact on calibration.

The results show the type of ensemble heuristic impacted transportability. The ensembles that performed the best in terms of discrimination when transported were the weighted fusion ensembles. The stacking ensemble did almost as well as the weighted fusion ensemble when there were sufficient labels, but it required labels in the new data it is being transported to whereas the weighted fusion ensembles did not. Requiring labels is a big disadvantage and therefore the weighted fusion ensembles are more useful. Interestingly, the simple mean fusion ensemble (uniform weighting) was comparable to the AUROC, age and database similarity weighted ensembles. Due to its simplicity, the mean fusion ensemble shows promise at being able to lead to more transportable prognostic models. If it worth noting, the age weighted ensembles may have benefitted in this study by the databases being similar (mostly US claims databases). For example, Optum claims is a mixture of patients that are similar to the patients in CCAE and MDCR, Therefore the age weighting may not perform well when the databases are more diverse. The weighted fusion ensembles and mixture of expert ensemble may have been impacted by the outcome rate differing between the databases. If the outcome is more common in a database, then a logistic regression model’s intercept is likely to be greater and the model’s mean predicted risk is likely to be higher than a model trained in data with fewer outcomes. This effectively may add more weighting to Level 1 models trained in databases that have a higher outcome percentage in the data.

The key advantage of this study is that we were able to compare the transportability of Level 1 models (developed in a single database) and ensembles combining Level 1 models developed in different databases across many prediction problems and across five datasets. In total we trained 21 (outcomes) × 5 (databases) single database models and created 21 (outcomes) × 5 (databases) × 7 (ensemble methods) ensemble models. The limitation of this study is the generalizability of findings as we only investigated one target population and we only used US data. In future work it would be useful to repeat this experiment across different target populations and externally validate the models (ensemble/non-ensemble) developed in this study across non-US databases. The OHDSI network and collaboration could be used to scale up this study across more diverse databases in future work [24]. In addition, there are numerous ways to combine the Level 1 models into an ensemble and we only investigated 7 simple approaches. However, these results provide a benchmark for comparing other ensembles techniques.

In this study 500,000 patients were sampled from each database (if there were more than 500,000 target population patients) as this provided a range of outcome sizes for the 21 outcomes investigated and enabled us to investigate the impact of outcome count in the study. Predicting rare outcomes is often an area of interest in healthcare and this may be where learning across multiple databases is more advantageous.

In future work it would be interesting to investigate whether rescaling the Level 1 models’ predictions within the ensemble, to make the mean predicted risk for each Level 1 model within the ensemble equal, could improve the weighted fusion or mixture of expert ensembles. Furthermore, it would be beneficial to investigate potential methods to recalibrate the ensembles given the calibration was shown to be poor. In addition, in this study we did not investigate pruning the Level 1 models within the ensembles, but this is an area of future research that may further improve transportability of an ensemble. In this study none of the Level 1 single database models achieved an AUROC ~ 0.5, but it may make sense to prune such models if the situation arises. Finally, we only investigated ensembles of LASSO logistic regression models. It would be interesting to repeat the experiment using different machine learning modeling methods such as logistic regression with Ridge or Elasticnet regularization.

## Conclusion

In this study we performed a large-scale empirical evaluation to investigate the transportability of a simple and feasible federated learning approach that uses ensemble learning to combine models developed independently in different databases. The results show that a mean fusion ensemble appears to transport to new data with higher discrimination compared to models developed in any single database. Consequently, developing a mean fusion ensemble of prognostic models developed using different databases (but for the same task) may lead to more clinically robust and useful prognostic models. However, recalibration is likely to be required.

## Supplementary Information

**Additional file 1.** Full details on the feature construction.

**Additional file 2.** Additional performance figures containing the AUROC confidence intervals and calibration intercept.

**Additional file 3.** Additional performance and model details: AUROC values per Level 1 and Level 2 model for each individual outcome plus the number of features in each Level 1 model.

## Data Availability

The data that support the findings of this study are available from Optum and IBM but restrictions apply to the availability of these data, which were used under license for the current study, and so are not publicly available. The IBM CCAE, MDCD and MDCR data that support the findings of this study are available from IBM MarketScan Research Databases (contact at: https://www.ibm.com/products/marketscan-research-databases/databases). The Optum data that support the findings of this study are available from Optum (contact at: https://www.optum.com/business/solutions/life-sciences/real-world-data.html).

## Abbreviations

AUROC: Area under the receiver operating characteristic curve
CCAE: IBM Commercial Claims and Encounters (database)
CDM: Common data model
HER: Electronic healthcare records
LASSO: Least absolute shrinkage and selection operator
MDCD: IBM Medicaid (database)
MDCR: IBM Medicare Supplemental Beneficiaries (database)
OHDSI: Observational Health Data Science and Informatics
OMOP: Observational medical outcomes partnership
US: United States
UK: United Kingdom
