# Supplementary material for: Learning patient-level prediction models across multiple healthcare databases: evaluation of ensembles for increasing model transportability
Source: BMC Med Inform Decis Mak. 2022 May 25;22:142. doi: 10.1186/s12911-022-01879-6 (PMC9134686; doi:10.1186/s12911-022-01879-6)
Supplement: Supplementary file 1 — Additional file 1. Full details on the feature construction. [file 12911_2022_1879_MOESM1_ESM.docx]

Appendix A – Feature construction

In this study we used the OHDSI FeatureExtraction R package to create features (<https://github.com/OHDSI/FeatureExtraction>). This R package contains SQL code that generates set of standard features for data in the OMOP CDM format. The standard features are generally binary that indicate whether a patient in the target population had a certain SNOMED (for conditions), RXNorm (for drugs) or other OMOP standard vocabularies during some time period prior to target population index. The package enables users to specify the table used to create the candidate features (e.g., ‘condition_occurrence’ or ‘drug_exposure’) and a period of time relative to target population index (e.g., between -365 days and -1 day relative to index).

For example, if ‘condition_occurrence between -30 days and 0 days relative to index’ are included as candidate features, then FeatureExtraction will find all the SNOMED codes that are recorded in the OMOP CDM ‘condition_occurrence’ table for any patient in the target population within 30 days prior to index and up to index (including index). For each of these SNOMED codes, e.g., ‘ABC’, a candidate feature is created called: ‘ABC condition_occurrence between -30 days and 0 days relative to index’. Any patient in the target population who had the code ‘ABC’ recorded within 30 days prior to index (including on index) will have a value of 1 for the candidate feature ‘ABC condition_occurrence between -30 days and 0 days relative to index’, otherwise the value is 0.

Many vocabularies have hierarchal structures, for example code A has children code such as A1, A2, A3 that are more specific medical conditions of code A. Some of the tables in the OMOP CDM aggregate the vocabulary codes using the hierarchy. For example, ‘drug_era’ aggregates up to drug ingredient. FeatureExtraction lets users use the hierarchy for conditions and drugs.

FeatureExtraction also extracts certain demographics (ethnicities, races, male/female, age in 5-year groups such as 0-4 years, 5-10 years, … 96-100 years) as binary features and the index month as a value from 1-12 (the month that the target population index occurred for each patient).

For this study we used the following features:

- Observations occurring between -30 days and 0 days relative to index
- Conditions (rolled up using hierarchy) occurring between -30 days and 0 days relative to index
- Drug ingredients occurring between -30 days and 0 days relative to index
- Visit type occurring between -30 days and 0 days relative to index
- Measurements occurring between -30 days and 0 days relative to index
- Procedures occurring between -30 days and 0 days relative to index
- Devices occurring between -30 days and 0 days relative to index
- Drug ingredients occurring between -365 days and 0 days relative to index
- Procedures occurring between -365 days and 0 days relative to index
- Measurements range occurring between -365 days and 0 days relative to index
- Measurements occurring between -365 days and 0 days relative to index
- Observations occurring between -365 days and 0 days relative to index
- Device occurring between -365 days and 0 days relative to index
- Visits occurring between -365 days and 0 days relative to index
- Conditions occurring between -365 days and 0 days relative to index
- Index month
- Ethnicity
- Age group (5 year bins)
- Race
- Gender
