# Supplementary material for: Learning patient-level prediction models across multiple healthcare databases: evaluation of ensembles for increasing model transportability
Source: BMC Med Inform Decis Mak. 2022 May 25;22:142. doi: 10.1186/s12911-022-01879-6 (PMC9134686; doi:10.1186/s12911-022-01879-6)
Supplement: Supplementary file 2 — Additional file 2. Additional performance figures containing the AUROC confidence intervals and calibration intercept. [file 12911_2022_1879_MOESM2_ESM.docx]

Appendix B – Additional Performance Figures


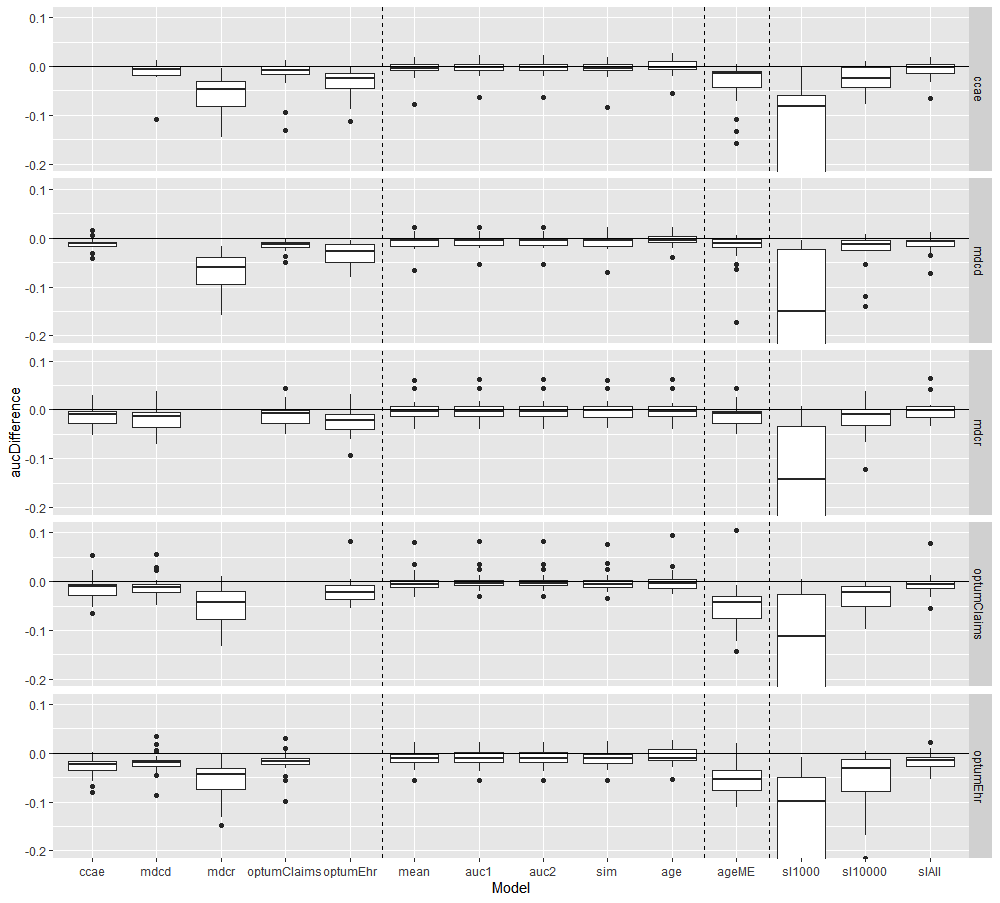


Figure B1 Zoomed in box plots showing the difference between the external validation AUROC minus the internal validation AUROC per non-ensemble (Level 1 model) and ensemble method (Level 2 model) across the five databases. The rows represent the external database (the database that was excluded from the model/ensemble development) that was used to fairly evaluate the models/ensembles. The x-axis represents the model/ensemble technique. Box plots centered around 0 with a small range indicate highly transportable and consistent external discriminative performance. The dashed vertical lines separate the non-ensembles, the fusion ensembles, the mixture of expert ensembles and the stacking ensembles.


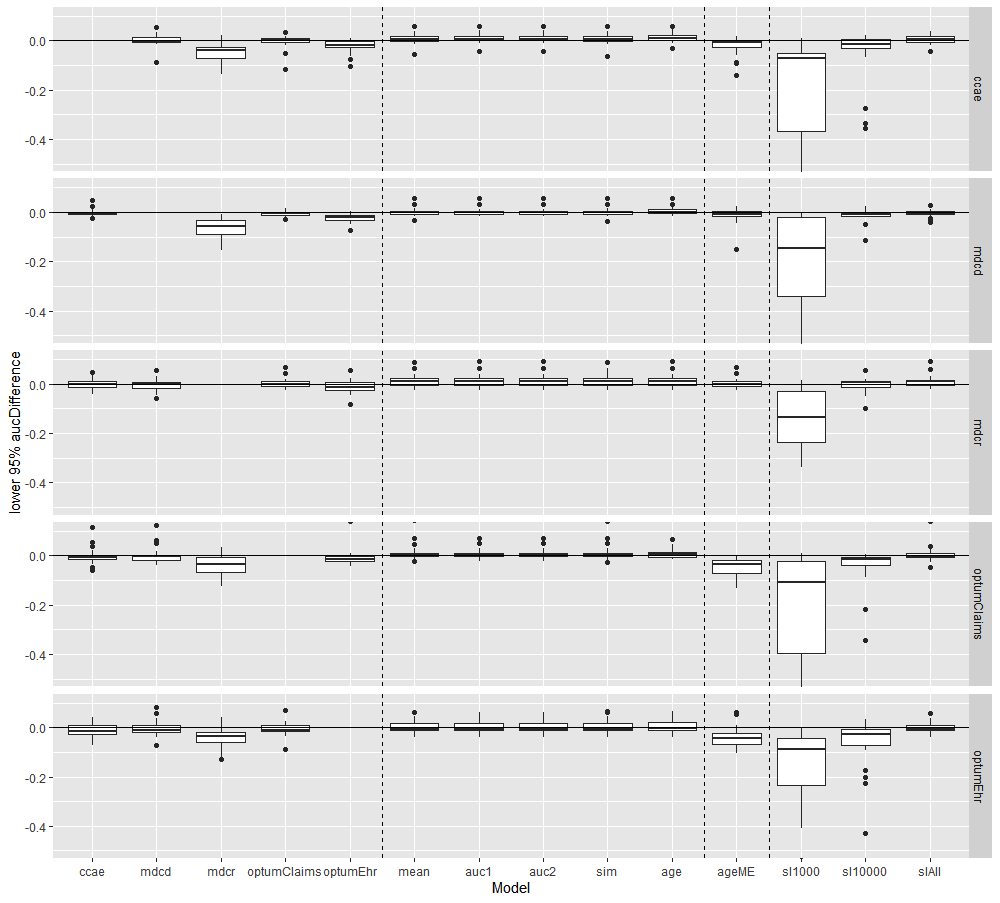


Figure B2 Box plots showing the difference between the external validation lower 95% confidence interval of the AUROC minus the internal validation lower 95% confidence interval of the AUROC per non-ensemble (Level 1 model) and ensemble method (Level 2 model) across the five databases. The rows represent the external database (the database that was excluded from the model/ensemble development) that was used to fairly evaluate the models/ensembles. The x-axis represents the model/ensemble technique. Box plots centered around 0 with a small range indicate highly transportable and consistent external discriminative performance. The dashed vertical lines separate the non-ensembles, the fusion ensembles, the mixture of expert ensembles and the stacking ensembles.


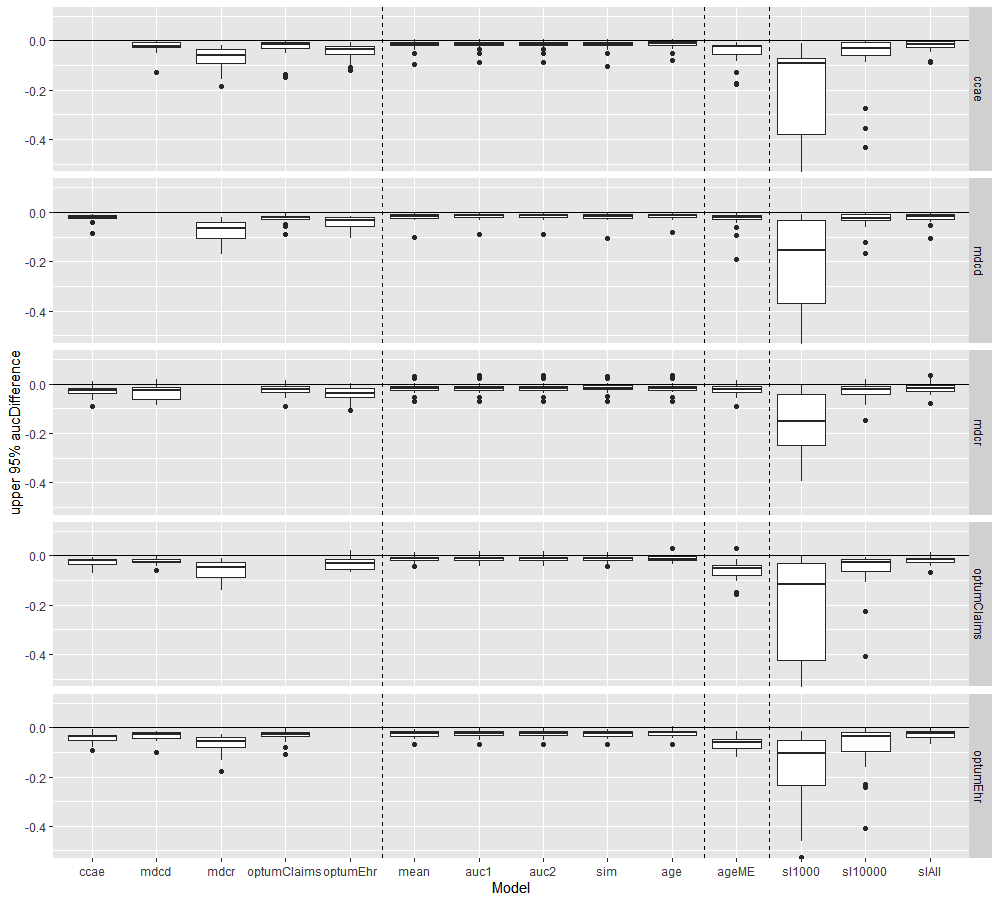


Figure B3 Box plots showing the difference between the external validation upper 95% confidence interval of the AUROC minus the internal validation upper 95% confidence interval of the AUROC per non-ensemble (Level 1 model) and ensemble method (Level 2 model) across the five databases. The rows represent the external database (the database that was excluded from the model/ensemble development) that was used to fairly evaluate the models/ensembles. The x-axis represents the model/ensemble technique. Box plots centered around 0 with a small range indicate highly transportable and consistent external discriminative performance. The dashed vertical lines separate the non-ensembles, the fusion ensembles, the mixture of expert ensembles and the stacking ensembles.


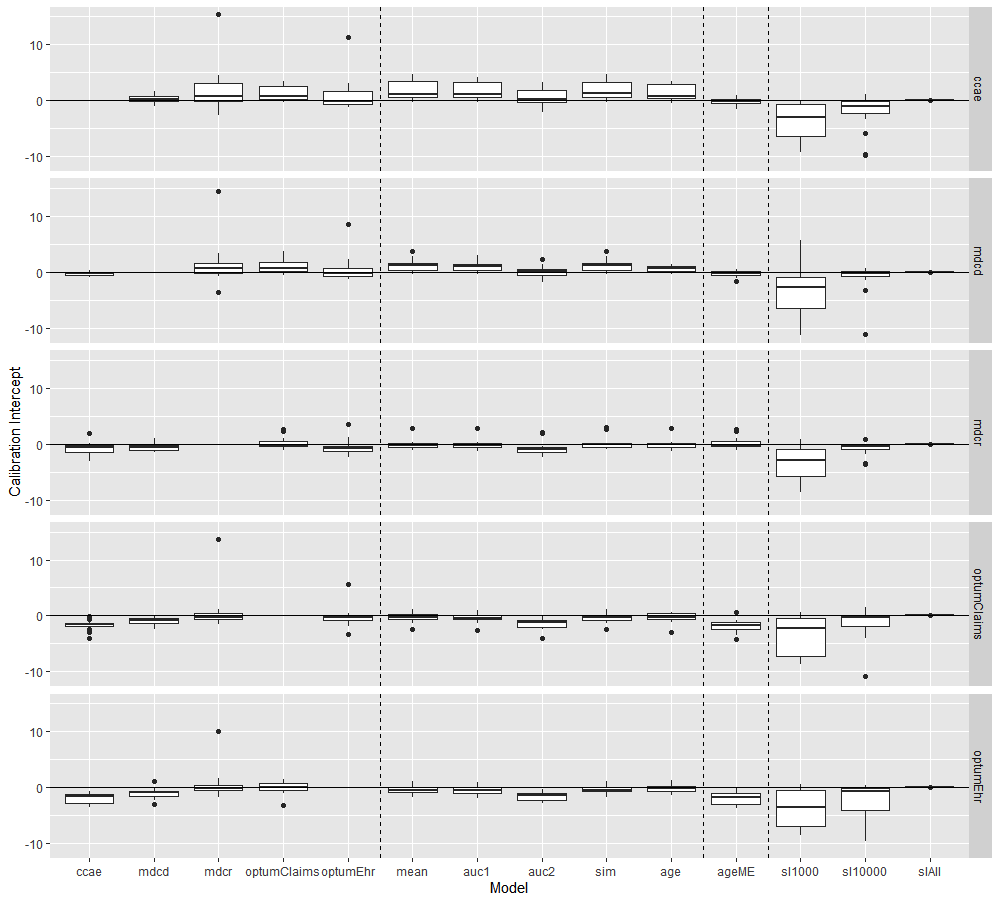


Figure B4 Box plots showing the external validation calibration intercept per non-ensemble (Level 1 model) and ensemble method (Level 2 model) across the five databases. The rows represent the external database (the database that was excluded from the model/ensemble development) that was used to fairly evaluate the models/ensembles. The x-axis represents the model/ensemble technique. Box plots centered around 0 with a small range indicate good calibration performance. The dashed vertical lines separate the non-ensembles, the fusion ensembles, the mixture of expert ensembles and the stacking ensembles.


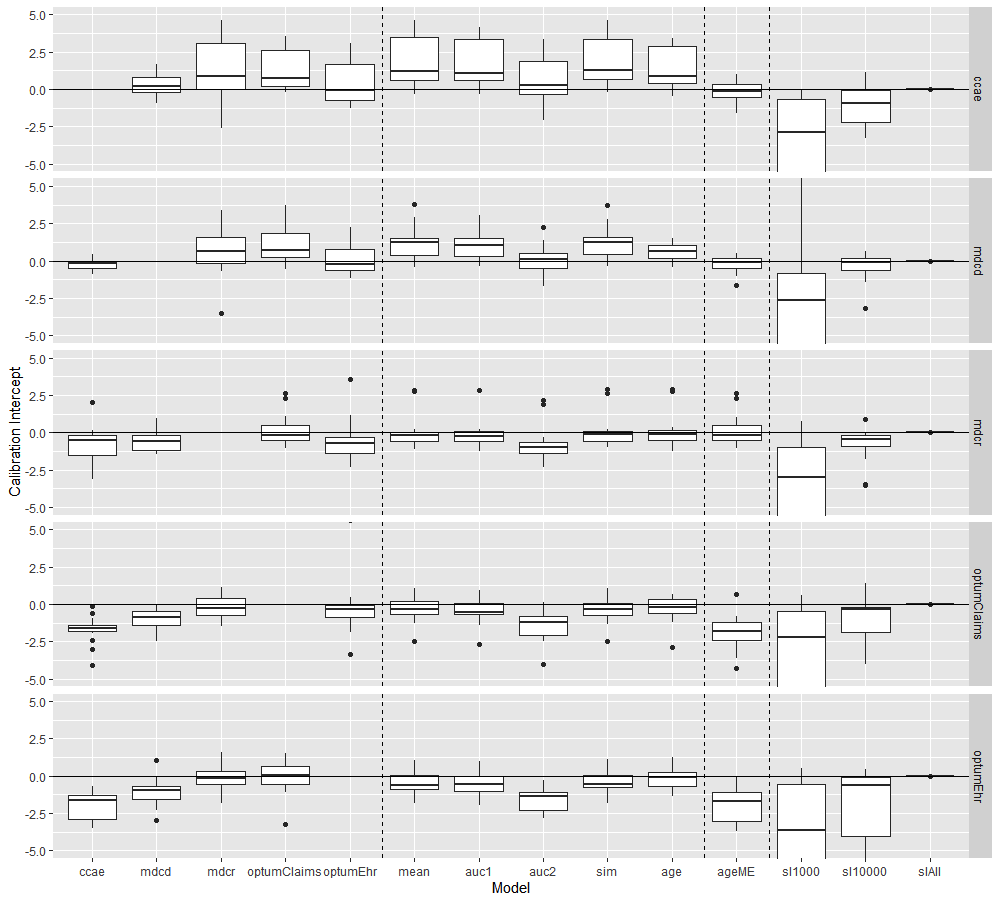


Figure B5 Zoomed in box plots showing the external validation calibration intercept per non-ensemble (Level 1 model) and ensemble method (Level 2 model) across the five databases. The rows represent the external database (the database that was excluded from the model/ensemble development) that was used to fairly evaluate the models/ensembles. The x-axis represents the model/ensemble technique. Box plots centered around 0 with a small range indicate good calibration performance. The dashed vertical lines separate the non-ensembles, the fusion ensembles, the mixture of expert ensembles and the stacking ensembles.
