# Supplementary material for: Learning patient-level prediction models across multiple healthcare databases: evaluation of ensembles for increasing model transportability
Source: BMC Med Inform Decis Mak. 2022 May 25;22:142. doi: 10.1186/s12911-022-01879-6 (PMC9134686; doi:10.1186/s12911-022-01879-6)
Supplement: Supplementary file 3 — Additional file 3. Additional performance and model details: AUROC values per Level 1 and Level 2 model for each individual outcome plus the number of features in each Level 1 model. [file 12911_2022_1879_MOESM3_ESM.docx]

Appendix C


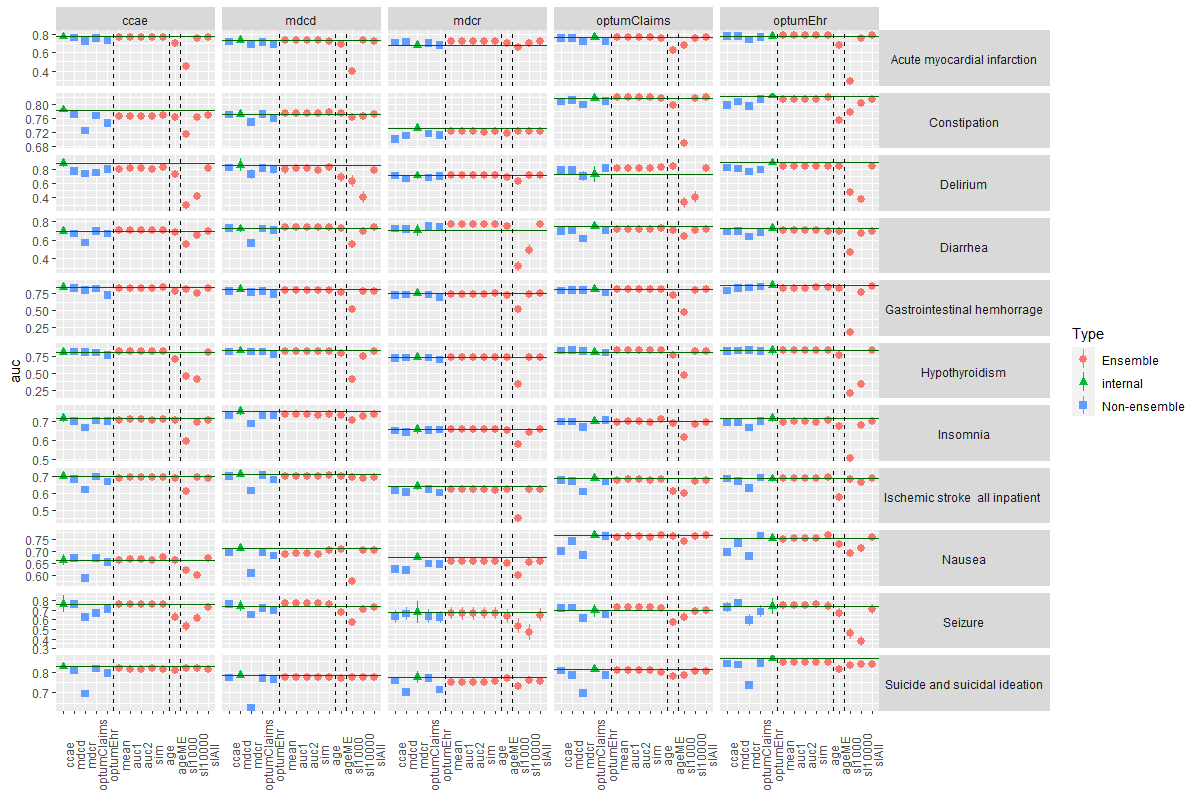
 Figure C1a - the discriminative performance in terms of AUROC of the database non-ensembles and the ensembles across validation databases (columns) and outcomes (rows). Green triangle points are the internal validation performance, blue square points correspond to non-ensemble models and red circle points correspond to ensembles. The 95% confidence intervals are marked as vertical lines.


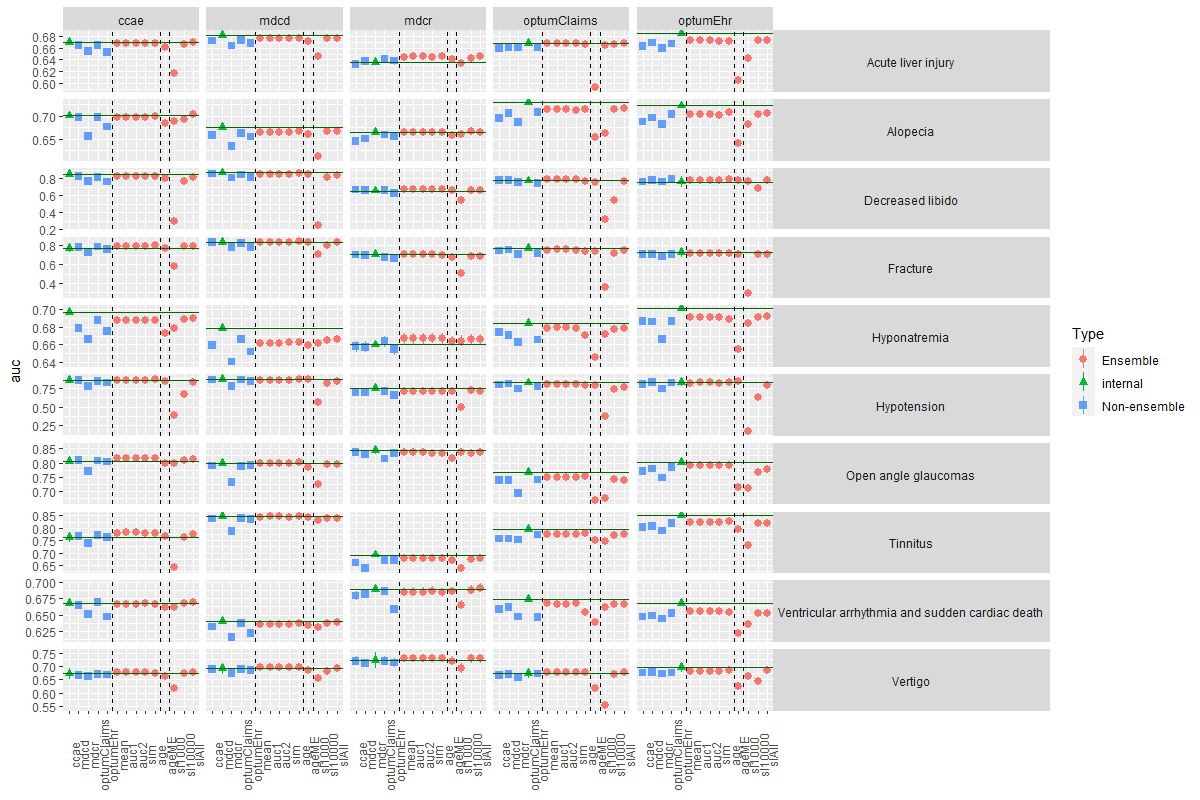


Figure C1b - the discriminative performance in terms of AUROC of the database non-ensembles and the ensembles across validation databases (columns) and outcomes (rows). Green triangle points are the internal validation performance, blue square points correspond to non-ensemble models and red circle points correspond to ensembles. The 95% confidence intervals are marked as vertical lines. The solid horizontal line is the internal validation performance. The dashed vertical lines divide the non-ensembles, the weighted ensembles, the mixture of expert ensemble and the stacker ensembles.

The internal validation performance is represented as the horizontal line and green triangle, this is the discriminative ability of a model developed and validated using the same database. The vertical dashed lines separate the non-ensembles, the weighted ensembles, the mixture of expert ensemble and the stacking ensembles. Figure A1a-A1b generally shows that the ensembles performed similarly to the best performing non-ensemble, except for the stacker trained using limited labelled data. The weighted ensembles all performed similarly and often discriminated equivalently or better than the model developed using the data (internal validation).

Number of predictors in each model:

| Outcome | CCAE | MDCR | MDCD | Optum EHR | Optum Claims |
| --- | --- | --- | --- | --- | --- |
| Acute liver injury | 327 | 164 | 462 | 832 | 738 |
| Acute myocardial infarction | 200 | 77 | 340 | 264 | 221 |
| Alopecia | 405 | 472 | 493 | 881 | 893 |
| Constipation | 353 | 430 | 524 | 899 | 861 |
| Decreased libido | 108 | 102 | 159 | 135 | 67 |
| Delirium | 73 | 88 | 94 | 145 | 48 |
| Diarrhea | 182 | 32 | 270 | 243 | 280 |
| Fracture | 233 | 139 | 159 | 130 | 89 |
| Gastrointestinal hemhorrage | 167 | 160 | 225 | 250 | 235 |
| Hyponatremia | 628 | 379 | 595 | 1009 | 820 |
| Hypotension | 162 | 195 | 176 | 127 | 318 |
| Hypothyroidism | 88 | 86 | 128 | 65 | 81 |
| Insomnia | 184 | 179 | 240 | 243 | 306 |
| Ischemic stroke all inpatient | 212 | 160 | 206 | 332 | 289 |
| Nausea | 259 | 282 | 309 | 518 | 443 |
| Open angle glaucomas | 362 | 309 | 348 | 652 | 663 |
| Seizure | 36 | 7 | 84 | 33 | 35 |
| Suicide and suicidal ideation | 591 | 211 | 961 | 728 | 464 |
| Tinnitus | 331 | 368 | 569 | 798 | 637 |
| Ventricular arrhythmia and sudden cardiac death | 616 | 458 | 504 | 636 | 793 |
| Vertigo | 161 | 71 | 215 | 214 | 177 |
